# Complete Genome Sequence of Clostridium innocuum Strain LC-LUMC-CI-001, Isolated from a Patient with Recurrent Antibiotic-Associated Diarrhea

**DOI:** 10.1128/MRA.00365-20

**Published:** 2020-07-09

**Authors:** K. E. Cherny, E. A. Ozer, T. J. Kochan, S. Johnson, L. K. Kociolek

**Affiliations:** a Ann & Robert H. Lurie Children’s Hospital of Chicago, Chicago, Illinois, USA; b Northwestern University Feinberg School of Medicine, Chicago, Illinois, USA; c Loyola University Chicago Stritch School of Medicine, Maywood, Illinois, USA; University of Maryland School of Medicine

## Abstract

Here, we report the complete genome sequence of Clostridium innocuum strain LC-LUMC-CI-001. As recently as 2018, *C. innocuum* was generally considered a benign gastrointestinal microorganism. This strain was isolated from the stool of a patient with recurrent Clostridioides difficile infection-like illnesses.

## ANNOUNCEMENT

Clostridioides difficile has been well established as the primary causal microorganism of antibiotic-associated diarrhea (AAD) (1, 2). Recently, a new association between Clostridium innocuum and AAD was reported in a retrospective study conducted in Taiwanese patients thought to have C. difficile infection (CDI) (3). Using 16S rRNA sequencing, *C. innocuum* but not C. difficile was identified in 5.5% of stools. *C. innocuum* isolates were frequently vancomycin resistant, cytotoxic to Vero and HT-29 cells and enteropathogenic using a mouse intestinal loop model, and caused pseudomembranous colitis in some patients (3).

Here, we report the complete genome sequence of *C. innocuum* strain LC-LUMC-CI-001, isolated from stool of a 48-year-old woman with multiply recurrent CDI. The Lurie Children’s Hospital Institutional Review Board (IRB) approved this project (IRB 2016-109). After one recurrence, she was successfully treated with fidaxomicin followed by a tapering, suppressive vancomycin regimen. Five days after restarting vancomycin, she developed marked watery, yellow stools, similar to prior CDI episodes. Stool was negative for C. difficile by PCR (Cepheid Xpert) and anaerobic culture. After being cultured on taurocholate-cefoxitin-cycloserine-fructose agar anaerobically for 48 h at 37°C, *C. innocuum* was confirmed by matrix-assisted laser desorption ionization–time of flight mass spectrometry. The isolate was vancomycin resistant (MIC, 48 μg/ml by Etest).

Genomic DNA (gDNA) for sequencing libraries was extracted using the BiOstic bacteremia DNA isolation kit (Qiagen, Germantown, MD) from an overnight culture grown in tryptic soy broth at 37°C under anaerobic conditions. The short-read library was prepared from gDNA using the Nextera XT kit (Illumina) and sequenced on the Illumina MiSeq platform to yield 855,510 pairs of 301-bp reads. Default parameters were used for all software unless otherwise specified. On-instrument adapter trimming using MiSeq Reporter v2.6 generated 329.3 Mb (76× genome coverage). A long-read nanopore library was prepared from gDNA using the ligation sequencing kit (Oxford Nanopore, UK; catalog number SQK-LSK109) and sequenced on the Oxford Nanopore MinION platform using a FLO-MIN106 flow cell. Base calling with default quality score filtering and demultiplexing of sequence reads was performed using Guppy v3.4.5, yielding 39,986 reads totaling 397.8 Mbp and an approximate genome coverage of 92×. The read *N*_50_ value was 15,274 nucleotides (nt) and the *L*_50_ value was 8,982 reads. Nanopore reads were assembled using Flye v2.6 using default parameters to generate a single circular 4,289,702-bp contig (4). Illumina reads were aligned to the assembly using BWA v0.7.17, and assembly errors were corrected using Pilon v1.23 with a –mindepth setting of 0.1 (5, 6). Serial read alignment and Pilon correction were performed 3 times until no further assembly corrections were generated. Pilon v1.23 using the “–fix all” option with default parameters except for a minimum depth (“–mindepth”) setting of 0.1 was used to identify, manually assess, and correct any residual homopolymer assembly errors (6). Circlator v1.5.5 was used to ensure circularization of the chromosome by trimming overlapping ends and rotating to the start of the *dnaA* gene (7). The final assembly contained a single chromosomal sequence of length 4,289,702 bp and a GC content of 43.9%. Annotation was performed using the NCBI Prokaryotic Genome Annotation Pipeline v4.11 (8, 9).

### Data availability.

This project has been deposited in DDBJ/ENA/GenBank under the accession number CP048837. The raw reads are available under the accession numbers SRX8107520 and SRX8107519 for Nanopore MinION and Illumina MiSeq, respectively. The version described here is the second version.
